# Prednisolone has a positive effect on the kidney but not on the liver of brain dead rats: a potencial role in complement activation

**DOI:** 10.1186/1479-5876-12-111

**Published:** 2014-05-02

**Authors:** Rolando Rebolledo, Bo Liu, Mohammed Z Akhtar, Petra J Ottens, Jian-ning Zhang, Rutger J Ploeg, Henri GD Leuvenink

**Affiliations:** 1 Department of Surgery, University Medical Center Groningen, Hanzeplein 1, 9713 GZ Groningen, The Netherlands; 2 Department of Surgery, Faculty of Medicine, University of Chile, Independencia 1027, 8380453 Santiago, Chile; 3 Department of Neurosurgery, Tianjin Medical University General Hospital, 154 Anshan Road, 300052 Tianjin, China; 4 Nuffield Department of Surgical Sciences, University of Oxford, Headington, OX3 9DU Oxford, UK

**Keywords:** Brain death, Organ donation, Steroids, Prednisolone, Kidney transplantation, Liver transplantation

## Abstract

**Background:**

Contradictory evidence has been published on the effects of steroid treatments on the outcomes of kidney and liver transplantation from brain dead (BD) donors. Our study aimed to evaluate this disparity by investigating the effect of prednisolone administration on BD rats.

**Methods:**

BD induction was performed in ventilated rats by inflating a Fogarty catheter placed in the epidural space. Prednisolone (22.5 mg/kg) was administered 30 min prior to BD induction. After four hours of determination of BD: serum, kidney and liver tissues samples were collected and stored. RT-qPCR, routine biochemistry and immunohistochemistry were performed.

**Results:**

Prednisolone treatment reduced circulating IL-6 and creatinine plasma levels but not serum AST, ALT or LDH. Polymorphonuclear influx assessed by histology, and inflammatory gene expression were reduced in the kidney and liver. However, complement component 3 (C3) expression was decreased in kidney but not in liver. Gene expression of HSP-70, a cytoprotective protein, was down-regulated in the liver after treatment.

**Conclusions:**

This study shows that prednisolone decreases inflammation and improves renal function, whilst not reducing liver injury. The persistence of complement activation and the negative effect on protective cellular mechanisms in the liver may explain the disparity between the effects of prednisolone on the kidney and liver of BD rats. The difference in the molecular and cellular responses to prednisolone administration may explain the contradictory evidence of the effects of prednisolone on different organ types from brain dead organ donors.

## Background

The shortage of organs for transplantation remains one of the most important issues facing the transplant community today. Increasingly, organs from brain dead extended criteria donors (ECD) and donors after circulatory arrest (DCD) are being used to address the organ deficit. The short and long term outcomes of allografts obtained from these donors are inferior when compared to living donors [1,2]. In the case of organs obtained from donors after brain death (DBD) it has been shown that HLA mismatched living donors have better outcomes even when cold ischemia times are taken into consideration [3]. The explanation for this lies in the process of brain death (BD) itself resulting in a non-physiological environment culminating in significant organ injury prior to organ procurement. The procurement, preservation and reperfusion phases of transplantation result in significant additional injury to the allograft rendering it susceptible short and long term dysfunction [4-6].

BD causes complex disturbances of normal homeostatic systems resulting in hemodynamic instability [7-10] hormonal impairment [11-13] and inflammation [14-17]. BD results in significant cerebral ischemia and intracranial hypertension resulting in parasympathetic activity followed by a severe vasoconstriction. This is due to an overriding sympathetic response and termed the “catecholamine storm” which is part of the Cushing reflex; a physiological response to maintain cerebral perfusion. A progressive paralysis of the spinal cord occurs, this cause the loss of vasomotor tone and leads to hemodynamic instability which characterizes this period [18-20].

Pituitary function is also affected following BD as adrenocorticotrophic hormone (ACTH) secretion is altered resulting in a transient rise in cortisol levels, which then diminishes progressing below baseline levels. In addition other hormones including T _4_/T _3_, anti-diuretic hormone (ADH) and insulin are reduced as a consequence of BD [21].

BD results in a heightened systemic inflammatory state as illustrated by the influx of polymorphonuclear neutrophils (PMNs) into kidney and liver tissues [15]. Systemic production of circulating cytokines including interleukin-6 (IL-6), interleukin-10 (IL-10), Tumor Necrosis Factor-alpha (TNF- *α*), Transforming Grown Factor-beta (TGF- *β*) and Monocyte chemotactic protein 1 (MCP-1) are thought to orchestrate this response. The trigger for this inflammatory process is not well understood, however recent evidence suggests there could be a role for cerebral cytokines that cross the blood-brain barrier, in addition to complement activation [22,23] and intestinal bacterial translocation [24,25].

A number of important considerations have been described for the ICU management of BD organ donors. There is general consensus about the importance of maintaining hemodynamic stability, but hormonal or anti-inflammatory treatments remains controversial despite promising experimental evidence [21]. The administration of glucocorticoids to donors has been used as hormonal replacement therapy with a varied degree of success [26-31].

In BD glucocorticoid administration could have several beneficial effects including anti-inflammatory properties and the ability to augment chrommaffin cells production of endogenous epinephrine [32]. The anti-inflammatory effects of steroids result from the pleiotropic interaction with the glucocorticoid receptor. The cortisol-glucocorticoid receptor complex can act through genomic and non-genomic downstream signalling pathways within the cell. This involves the dissociation of heat-shock proteins and the interaction with membrane-associated receptors, second messengers and activation of transcription factors such as Nuclear factor kappa-light-chain-enhancer of activated B cells (NF- *κ*B) [33].

We hypothesize that prednisolone pre-treatment of BD rats could reduce the inflammatory response and improve the quality of kidney and liver allografts. The aim of this study is to assess the potential benefit of the prednisolone pre-treatment in the liver and kidney of BD rats.

## Methods

### Animals

Male adult Fisher F344 rats (250 - 300 g) were used in all experiments. All animals received care in compliance with the guidelines of the local animal ethics committee according to Experiments on Animals Act (1996) issued by the Ministry of Public Health, Welfare and Sports of the Netherlands. Brain death (BD) was induced as follows: animals were anesthetized using isoflurane with O2. A cannula was inserted in the femoral artery and vein for continuous mean arterial pressure (MAP) monitoring and fluid administration. Animals were intubated via a tracheostomy and ventilated throughout the experiment. A no. 4 Fogarty catheter (Edwards Lifesciences Co, Irvine, CA) was placed in the epidural space through a frontolateral burr hole, and slowly inflated (0.16 ml/min) with saline using a syringe pump (Terufusion, Termo Co, Tokyo, Japan). Inflation of the balloon was terminated once the MAP began to improve after a characteristic period of hypotension, reflecting the autonomic storm. BD was confirmed by the absence of corneal and pupillary reflexes and a positive apnea test. Following confirmation of BD, anesthesia was terminated but ventilation continued. MAPs were maintained above 80mmHg using Hydroxyethyl starch (HAES) 10% (Fresenius Kabi AG, Bad Homburg, Germany) with a maximum rate of 1 ml/hr. If HAES was insufficient to maintain the MAP, noradrenaline (NA) 0.01 mg/ml was administered. A homeothermic blanket control system was used throughout the BD maintenance period. Four hours after determination of BD, rats were heparinized with 500 IU heparin. A laparotomy was subsequently performed and blood collected from the aorta. Organs were flushed with 0.9% saline and snap frozen in liquid nitrogen and collected plasma stored at −80°C.

Rats were randomly assigned to each group. The animal number was calculated using the method of Russ Lenth [34], with a meaningful difference of 50%, a variability (sigma) of 0.3 and a power of 0.9. Sham-operated rats served as controls and were ventilated for half an hour under anesthesia before termination. This was in accordance with the requirement of our local Animal Welfare Committee guidance for the use of sham controls in experiments. Prednisolone or saline was administered intravenously 30 minutes before the start of BD induction. Prednisolone dosage was chosen based on previous experiments and to give the best hemodynamic stability (22.5 mg/kg).

The following experimental groups were established: 

• Sham-operated animals receiving saline (n = 8).

• Sham-operated animals receiving Prednisolone (n = 8).

• BD animals receiving saline (n = 8).

• BD animals receiving Prednisolone (n = 8).

### Plasma determinations

Plasma creatinine was determined in the biochemistry lab of the University Medical Center Groningen. The level of IL-6 in the plasma was determined by a rat enzyme-linked immunosorbent assay (IL-6 ELISA) kit (R&D Systems Europe Ltd. Abingdon, Oxon OX14 3NB, UK), according to the manufacturer instructions. All samples were analyzed in duplicate and read at 450 nm.

### RNA isolation and cDNA synthesis

Total RNA was isolated from whole kidneys and liver sections by using TRIzol (Life Technologies, Gaithersburg, MD). RNA samples were verified for absence of genomic DNA contamination by performing RT-PCR reactions in which the addition of reverse transcriptase was omitted, using Glyceraldehyde 3-phosphate dehydrogenase (GAPDH) primers. For cDNA synthesis, 1 *μ*l T11VN Oligo-dT (0.5 *μ*g/ *μ*l) and 1 *μ*g mRNA were incubated for 10 min at 70°C and cooled directly after that. cDNA was synthesized by adding a mixture containing 0.5 *μ*l RnaseOUT Ribonuclease inhibitor (Invitrogen, Carlsbad, USA), 0.5 *μ*l RNase water (Promega), 4 *μ*l 5 x first strand buffer (Invitrogen), 2 *μ*l DTT (Invitrogen), 1 *μ*l dNTPO’s and 1 *μ*l M-MLV reverse transcriptase (Invitrogen, 200U). The mixture was held at 37°C for 50 min. Next, reverse-transcriptase was inactivated by incubating the mixture for 15 min at 70°C. Samples were stored at −20°C.

### Real-Time PCR

Fragments of several genes were amplified with the primer sets outlined in Table 1. Pooled cDNA obtained from brain-dead rats were used as internal references. Gene expression was normalized with the mean of *β*-actin mRNA content. Real-Time PCR was carried out in reaction volumes of 15 *μ*l containing 10 *μ*l of SYBR Green mastermix (Applied biosystems, Foster City, USA), 0.4 *μ*l of each primer (50 *μ*M), 4.2 *μ*l of nuclease free water and 10 ng of cDNA. All samples were analyzed in triplicate. Thermal cycling was performed on the Taqman Applied Biosystems 7900HT Real Time PCR System with a hot start for 2 min at 50°C followed by 10 min 95°C. Second stage was started with 15 s at 95°C (denaturation step) and 60 s at 60°C (annealing step and DNA synthesis). The latter stage was repeated 40 times. Stage 3 was included to detect formation of primer dimers (melting curve) and begins with 15 s at 95°C followed by 60 s at 60°C and 15 s at 95°C. Primers were designed with Primer Express software (Applied Biosystems) and primer efficiencies were tested by a standard curve for the primer pair resulting from the amplification of serially diluted cDNA samples (10 ng, 5 ng, 2.5 ng, 1.25 ng and 0.625 ng) obtained from brain-dead rats. PCR efficiency were found to be 1.8<*ε*<2.0. Real-time PCR products were checked for product specificity on a 1.5% agarose gel. Results were expressed as 2- △△ CT (CT: Threshold Cycle).

**Table 1:** Primer sequences used for Real-Time PCR

**Gene**	**Primers**	**Amplicon size (bp)**
TNF- *α*	5’-GGCTGCCTTGGTTCAGATGT-3’ 5’-CAGGTGGGAGCAACCTACAGTT-3’	79
IL-1 *β*	5’- CAGCAATGGTCGGGACATAGTT-3’ 5’-GCATTAGGAATAGTGCAGCCATCT-3’	75
IL-6	5’-CCAACTTCCAATGCTCTCCTAATG-3’ 5’-TTCAAGTGCTTTCAAGAGTTGGAT-3’	89
C3	5’-CAGCCTGAATGAACGACTAGACA-3’ 5’-TCAAAATCATCCGACAGCTCTATC-3’	.96
MCP-1	5’-CTTTGAATGTGAACTTGACCCATAA-3’ 5’-ACAGAAGTGCTTGAGGTGGTTGT -3’	78
HO-1	5’-CTCGCATGAACACTCTGGAGAT-3’ 5’-GCAGGAAGGCGGTCTTAGC-3’	74
HSP-70	5’-GGTTGCATGTTCTTTGCGTTTA-3’ 5’-GGTGGCAGTGCTGAGGTGTT-3’	80
BAX	5’-GCGTGGTTGCCCTCTTCTAC-3’ 5’-TGATCAGCTCGGGCACTTTAGT-3’	74
BCL-2	5’-CTGGGATGCCTTTGTGGAA-3’ 5’-TCAGAGACAGCCAGGAGAAATCA-3’	70

### Immunohistochemistry

To detect polymorphonuclear cells in kidney and liver, immunohistochemistry was performed on 5- *μ*m tissue cryosections. Sections were fixated for 10 min using acetone. Next, sections were stained with HIS-48 mAb (supernatant, two times diluted) using an indirect immunoperoxidase technique. Endogenous peroxidase was blocked using H _2_*O*_2_ 0.01% in phosphate-buffered saline for 30 mins. After thorough washing, sections were incubated with horseradish peroxidase-conjugated rabbit anti-mouse IgG as a secondary antibody for 30 mins, followed by goat anti-rabbit IgG as a tertiary antibody for 30 mins (both from Dako, Glostrup, Denmark). The reaction was developed using 9-amino-ethylcarbazole as chromogen and H _2_*O*_2_ as substrate. Sections were counterstained using Mayer hematoxylin solution (Merck, Darmstadt, Germany). Negative antibody controls were performed. Localization of immunohistochemical staining was assessed by light microscopy. For each tissue section, positive cells per field were counted in 10 microscopic fields of the tissue at 40x magnification. Results were presented as number of positive cells per glomerulus in the kidney and number of positive cells per field in the liver.

### Statistical analysis

Statistical analyses were performed using Prism 5.0. GraphPad. The two-way ANOVA test was performed, followed by the Bonferroni post-test. Specific analysis for the use of HAES and NA between groups was evaluated using the Mann-Whitney test. The significance level was set at a p value of < 0.05. Results are presented as mean ± SD (standard deviation).

## Results

**Figure 1:**
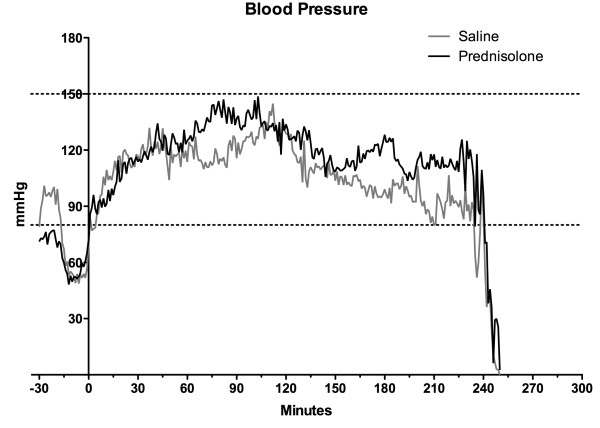
**Blood pressure registry.** The record started with the brain death induction, we considered the time “0” as the end of brain death induction and the starting of BD period. As a black-continuous line the mean in each minute of blood pressure for the prednisolone brain death group. As a grey-continuous line the mean in each minute for the saline brain death group.

Induction of BD showed a consistent and uniform pattern in alterations of the MAP, with a mean time to declaration of BD of 31.6 ± 2.73 minutes after commencing balloon inflation. All 32 animals were kept with a MAP above 80 mmHg during the experiment (Figure 1), after the confirmation of BD. In saline treated group, infusion of HAES 10% (2.7 ml ± 1.1) was needed to maintain MAP whereas in the prednisolone treated group significantly less was required (1.3 ml ± 1.3 HAES 10%). Administration of NA was comparable between both groups (saline: 0.11 ± 0.12 mg, prednisolone: 0.05 ± 0.09 mg).

**Figure 2:**
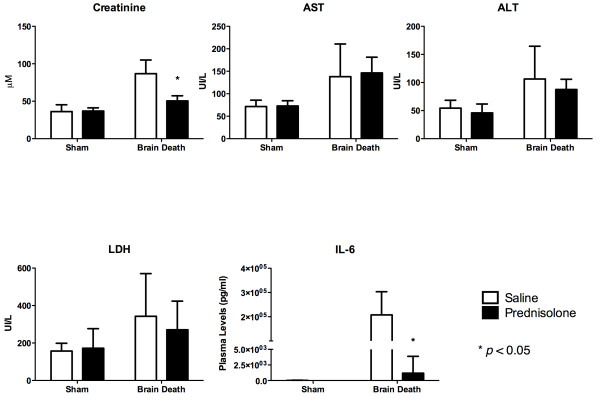
**Plasmatic levels of kidney function markers and liver injury markers.** Creatinine (Sham operated + Saline: 36 ± 9.24 *μ*M; Sham operated + prednisolone treatment: 36.88 ± 4.29 *μ*M; BD + Saline 86.63 ± 18.37 *μ*M; BD + prednisolone treatment: 50.38 ± 6.91 *μ*M), AST (Sham operated + Saline: 71.50 ± 14.19 U/l; Sham operated + prednisolone treatment: 72.88 ± 11.66 U/l; BD + Saline: 138.0 ± 72.76 U/l; BD + prednisolone treatment: 146.3 ± 35.07 U/l), ALT (Sham operated + Saline: 54.38 ± 13.97 U/l; Sham operated + prednisolone treatment: 45.88 ± 15.82 U/l; BD + Saline: 106.3 ± 58.38 U/l; BD + prednisolone treatment: 87.50 ± 18.33 U/l), LDH (Sham + Saline: 157.1 ± 41.54 U/l; Sham + prednisolone treatment: 172.4 ± 104.2 U/l; BD + Saline: 342.8 ± 228.0 U/l; BD + prednisolone treatment: 270 ± 152.9 U/l) and IL-6 (Sham operated + Saline: 15.63 ± 29.69 pg/ml; Sham operated + prednisolone: undetectable; BD + Saline: 2.07 × 10 ^5^± 9.59 × 10 ^4^ pg/ml; BD + prednisolone treatment: 1.18 × 10 ^3^± 2.69 × 10 ^3^ pg/ml).

Plasma creatinine levels was significantly different between sham and BD animals treated with saline solution. Creatinine plasma levels in BD animals treated with prednisolone (50.38 ± 6.90) was significantly lower compared to BD rats pre-treated with saline (86.63 ± 18.37) (Figure 2).

Assessing cellular liver injury, aspartate aminotransferase (AST), alanine aminotransferase (ALT) and lactate dehydrogenase (LDH) plasma levels were significantly increased after BD in comparison to sham animals. No significant differences were found in AST, ALT or LDH plasma levels between prednisolone or saline treated BD rats (Figure 2).

Our results demonstrated a significant decrease in IL-6 levels in BD animals treated with prednisolone compared to BD animals treated with saline (Figure 2).

**Figure 3:**
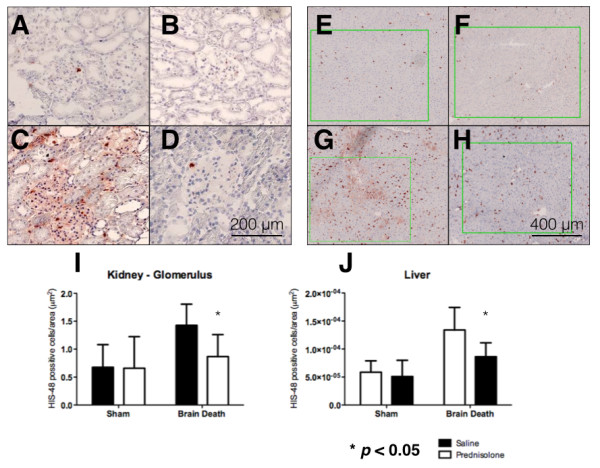
**PMN infiltration quantification and stanning.** Kidneys from **A)** Sham operated + Saline, **B)** Sham operated + prednisolone treatment, **C)** BD + Saline, **D)** BD + prednisolone treatment rats. PMN infiltration in livers from **E)** Sham operated + Saline, **F)** Sham operated + prednisolone treatment, **G)** BD + Saline, **H)** BD + prednisolone treatment rats, 40x magnification. **I)** Quantification of PMN infiltration in kidney and **J)** liver.

Tissue cryosections were stained with HIS-48 mAb to identify PMNs. The number of positive cells per glomerulus in the kidney was 0.67 ± 0.4 in sham-operated controls but was elevated to 1.42 ± 0.4 positive cells in the BD + saline group. Treatment with prednisolone reduced the PMN influx to 0.86 ± 0.4 positive cells, reaching statistical significance compared to the BD saline group (Figure 3).

In the case of the liver, the number of positive cells per microscopic field was 5.85 ± 2.0 in sham-operated controls but was elevated to 13.41 ± 4.0 positive cells in the BD + saline group. Treatment with prednisolone significantly diminished the PMN influx to 6.97 ± 2.5 positive cells (Figure 3).

**Figure 4:**
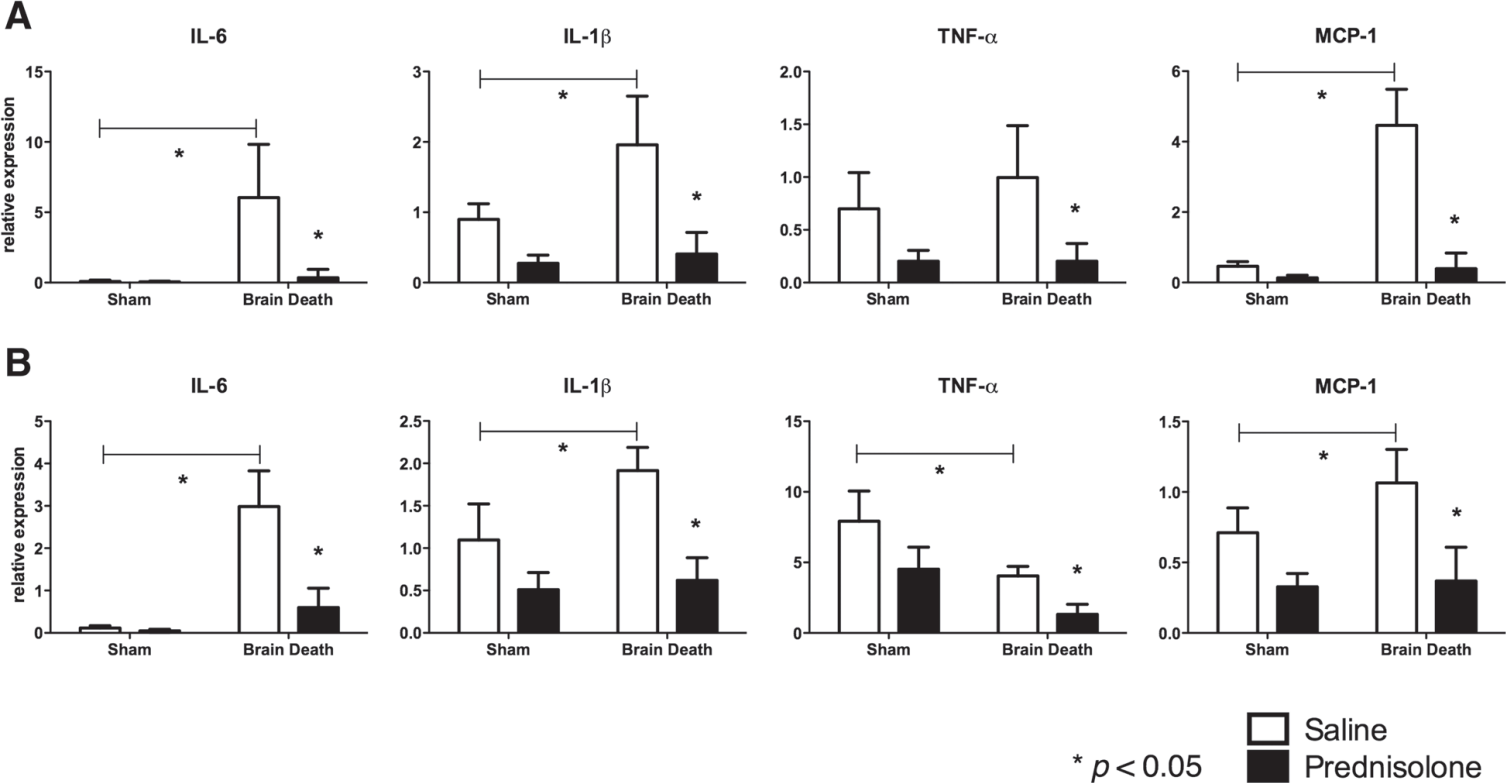
**Relative expression of inflammatory genes.A)** Kidney samples. IL-6 (Sham operated + Saline: 0.08 ± 0.09; Sham operated + prednisolone treatment: 0.05 ± 0.05; BD + Saline: 6.04 ± 3.79; BD + prednisolone treatment: 0.34 ± 0.59), IL-1 *β* (Sham operated + Saline: 0.89 ± 0.22; Sham operated + prednisolone treatment: 0.27 ± 0.11; BD + Saline: 1.95 ± 0.69; BD + prednisolone treatment: 0.40 ± 0.30), TNF- *α* (Sham operated + Saline: 0.69 ± 0.34; Sham operated + prednisolone treatment: 0.20 ± 0.10; BD + Saline: 0.99 ± 0.49; BD + prednisolone treatment: 0.20 ± 0.16), MCP-1 (Sham operated + Saline: 0.46 ± 0.13; Sham operated + prednisolone treatment: 0.13 ± 0.07; BD + Saline 4.46 ± 1.02; BD + prednisolone treatment: 0.39 ± 0.44). **B)** Liver samples. IL-6 (Sham operated + Saline: 0.11 ± 0.05; Sham operated + prednisolone treatment: 0.04 ± 0.03; BD + Saline: 2.98 ± 0.84; BD + prednisolone treatment: 0.60 ± 0.45), IL-1 *β* (Sham operated + Saline: 1.09 ± 0.42; Sham operated + prednisolone treatment: 0.50 ± 0.20; BD + Saline: 1.91 ± 0.27; BD + prednisolone treatment: 0.61 ± 0.26), TNF- *α* (Sham operated + Saline: 7.91 ± 2.13; Sham operated + prednisolone treatment: 4.51 ± 1.57; BD + Saline: 4.04 ± 0.66; BD + prednisolone treatment: 1.3 ± 0.71), MCP-1 (Sham operated + Saline: 0.71 ± 0.17; Sham operated + prednisolone treatment: 0.32 ± 0.09; BD + Saline: 1.06 ± 0.23; BD + prednisolone treatment: 0.36 ± 0.24).

qRT-PCR evaluation of gene expression revealed significantly upregulated IL-6, Interleukin 1 beta (IL-1 *β*) and monocyte chemoattractant protein-1 (MCP-1) in kidneys as a consequence of BD compared to sham controls. Relative expression of IL-6, IL-1 *β*, MCP-1 was significantly reduced in the prednisolone treated groups in the kidney samples (Figure 4).

In liver, relative expression of IL-6, IL-1 *β* and MCP-1 was significantly increased following BD. Prednisolone treatment significantly reduced IL-6, IL-1 *β* and MCP-1 compared to the BD control group (Figure 4).

TNF- *α* is a monocyte derived cytotoxin implicated in mediating apoptosis. In the kidney the relative expression of TNF- *α* increased but not to a significant level following BD. TNF- *α* was significantly down-regulated following prednisolone pre-treatment. However, in the liver the relative expression of TNF- *α* was significantly down-regulated following BD and further down-regulated following prednisolone treatment in the BD group (Figure 4).

**Figure 5:**
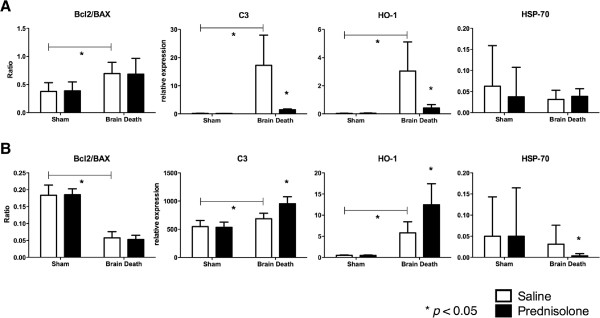
**Relative expression of C3, HO-1 and HSP-70.** Ratio of Bcl2/BAX expression. **A)** Kidney samples. C3 (Sham operated + Saline: 0.18 ± 0.04; Sham operated + prednisolone treatment: 0.16 ± 0.02; BD + Saline: 17.27 ± 10.74; BD + prednisolone treatment: 1.47 ± 0.26), HO-1 (Sham operated + Saline: 0.03 ± 0.01; Sham operated + prednisolone treatment: 0.04 ± 0.01; BD + Saline: 3.05 ± 2.06; BD + prednisolone treatment: 0.42 ± 0.23), HSP-70 (Sham operated + Saline: 0.06 ± 0.09; Sham operated + prednisolone treatment: 0.03 ± 0.07; BD + Saline: 0.03 ± 0.02; BD + prednisolone treatment: 0.03 ± 0.01), Ratio Bcl2/BAX (Sham operated + Saline: 0.37 ± 0.15; Sham operated + prednisolone treatment: 0.38 ± 0.15; BD + Saline: 0.69 ± 0.19; BD + prednisolone treatment: 0.69 ± 0.28). **B)** Liver samples. C3 (Sham operated + Saline: 546.9 ± 109.4; Sham operated + prednisolone treatment: 534.2 ± 92.18; BD + Saline: 687.4 ± 96.13; BD + prednisolone treatment: 952.2 ± 125.3), HO-1 (Sham operated + Saline: 0.47 ± 0.11; Sham operated + prednisolone treatment: 0.45 ± 0.13; BD + Saline: 5.78 ± 2.65; BD + prednisolone treatment: 12.47 ± 4.96), HSP-70 (Sham operated + Saline: 0.05 ± 0.09; Sham operated + prednisolone treatment: 0.05 ± 0.11; BD + Saline: 0.03 ± 0.04; BD + prednisolone treatment: 0.003 ± 0.005), Ratio Bcl2/BAX (Sham operated + Saline: 0.18 ± 0.03; Sham operated + prednisolone treatment: 0.18 ± 0.01; BD + Saline: 0.05 ± 0.01; BD + prednisolone treatment: 0.05 ± 0.01).

In the kidney C3 expression was increased following BD and attenuated following prednisolone treatment. While in the liver, C3 expression increased following BD and further increased due to prednisolone treatment (Figure 5).

To assess the effect on apoptotic pathways we studied the B-cell lymphoma 2 (Bcl2)/Bcl-2 associated X protein (BAX) ratio. In the kidney this ratio was increased following BD but decreased in the liver. Prednisolone treatment did not significantly modify this ratio under any experimental condition (Figure 5).

We studied the effects of the interventions on the relative expression of two cytoprotective genes. The relative expression of Heme oxygenase 1 (HO-1) in the kidney was up-regulated following BD and down-regulated following pre-treatment with prednisolone. In the liver the HO-1 expression was significantly up-regulated following prednisolone treatment. The relative HSP-70 expression in the kidney was not affected by BD nor prednisolone treatment. However, the relative expression in the liver was significantly down-regulated due to prednisolone pre-treatment (Figure 5).

## Discussion

Disparity exists in the literature with regards to the beneficial effects of prednisolone administration to the donor on the outcomes of solid organ transplantation. Multiple large randomized control trials (RCT) have evaluated the effects on steroid administration on the outcomes of lung, kidney and liver transplant [35-37]. In a RCT, Bonser et al. evaluated the effects of 1g of methylprednisolone administration to BD organ donors 7 hours prior to lung explantation, demonstrating no effect on increasing lung yields [29]. Others in the realms of kidney transplantation have also failed to detect a significant improvement in the kidney survival at three months when 5g of methylprednisolone is administered to donors 2-4 hours prior to explantation. The only clinical trial to suggest a positive outcome was performed by Kotsch et al. demonstrating a significant effect on down-regulation of pro-inflammatory cytokines and reduced incidence of acute rejection [28]. In contrast, an Austrian study failed to identify a benefit of 1g of methylprednisolone administration 6 hours prior to liver recovery [38].

Despite the heterogeneity of outcomes of these trials, steroid administration has, in many countries, been incorporated into donor management protocols. A review by Bugge et al. suggests the routine administration of methylprednisolone in all DBDs [39]. Indeed in the UK methylprednisolone is being trialed as part of a national donor optimization care-bundle pathway. Overall there is a clear need to establish the effects of steroid treatment in the donor and evaluate the potential differential effects of treatment on different organ systems.

This study was designed as a proof of concept study to evaluate the differential effects of prednisolone administration to brain dead rats. The results of our study suggest that the effect of steroid therapy in the donor will have differential effects on different organ systems. Our study confirms that steroid administration can improve the hemodynamic stability of the BD rodent. We hypothesize this effect occurs through facilitating the retention of sodium and water, and increasing the release of endogenous epinephrine [40]. Outside of this study, donor hemodynamic stability has been shown to reduce pre-procurement injury to allografts and improve short and long-term outcomes [41,42].

Our study has also shown that steroid administration does have an anti-inflammatory effect as illustrated by the reduction of circulating IL-6. IL-6 has been shown to be significantly up-regulated following BD and is hypothesized to be a key cytokine mediating the inflammatory cellular response to BD through an NF *κ*B mediated pathway with the downstream effectors being selectin, MCP-1 and TNF- *α*[43]. Indeed authors speculate that the link between cerebral injury and peripheral organ injury lies in an IL-6 mediated inflammatory response.

In our study prednisolone had different and disparent effects on kidney and liver allografts. In the kidney we demonstrated a significant improvement on kidney function, as assessed by serum creatinine and a reduction in infiltrating PMNs. This was accompanied by a reduction in expression of IL-6, MCP1 and IL-1 *β*, we demonstrated a reduction of TNF- *α* expression following methylprednisolone administration although this did not reach significance. We were able to demonstrate a reduction in C3 expression, having previously demonstrated the potential detrimental effects of complement activation in the BD donor [22,44,45]. We believe these anti-inflammatory effects, in conjunction with the hemodynamic stability conferred by prednisolone administration reduces the injury to the kidney and result in the improved kidney function.

Our study also demonstrated a reduction in HO-1 expression in the kidney following methylprednisolone pretreatment of BD rats. As has been shown by Terry et al. [46] HO-1 is up-regulated by inflammatory cytokines like IL-1 *α* via protein kinase C and phospholipase A2 in endothelial cells. We hypothesize that prednisolone is down-regulating HO-1 in the kidney by reducing the expression of inflammatory cytokines. In contrast to the kidney, in the liver HO-1 expression was increased following prednisolone pretreatment of the brain dead rat, even when the expression of inflammatory genes was decreased. We think that these results could be an indication of the cellular stress during the brain death period.

In the liver we failed to detect a significant effect of prednisolone pre-treatment of the BD rat to reduce hepatocyte injury as assessed by AST and ALT, we did however demonstrate a significant effect on reducing PMN infiltration and IL-6, MCP-1 and IL-1 *β* expression. We found that TNF- *α* expression was reduced in the liver following BD in comparison to sham operated animals. This effect might be related with the surgical stress in both groups and could be explain by a shorter timeframe between the surgical procedure and the organ retrieval in sham operated animals. However, TNF- *α* expression was further reduced in the prednisolone pretreated BD rats compare to the saline pretreated BD animals. TNF- *α* affects cell cycle and apoptotic pathways through NF *κ*B and MAP kinase mediated pathways. Our previous work has suggested TNF- *α* expression is increased following BD in the liver and systemically. We have previously been able to reduce systemic TNF- *α* release by stimulating the vagus nerve in a rodent model of brain death [47]. Concurrently Kotch et al. demonstrated pre-treatment of human donors with methylprednisolone could reduce TNF- *α* expression, supporting our findings.

Our study demonstrated relatively less apoptosis in the liver compared to the kidney following BD as demonstrated by the Bcl2/Bax. The comparatively low levels of TNF- *α* in the liver following BD may in part explain this. However, prednisolone failed to have a further effect on reducing the Bcl2/Bax ratio.

In contrast to the kidney, HSP70 expression in the liver following BD and prednisolone pre-treatment was down-regulated. Stoot et al. [48] showed that HSP70 activity could prevent C3 expression. Interestingly, our study demonstrated an increase in C3 expression in the liver following BD and prednisolone pre-treatment. It is likely that prednisolone is reducing HSP70 expression in the liver but not in the kidney and thereby increase or facilitate C3 deposition in the liver. This may explain the lack of prednisolone effect on reducing liver injury.

## Conclusion

There are a number of differences in the way organs respond to BD and donor treatment. We have demonstrated that the administration of prednisolone as a pre-treatment agent to the BD donor is likely to have differential effects on the kidney and the liver. The lack of effect of prednisolone in our experimental set up could in part, be due to the complement mediated injury. We acknowledge that we have not evaluated the effect of prednisolone after BD induction or time-course effect of prednisolone therapy at earlier or later time-points during the BD period. We also have not evaluated the effect of compounding the injury during the donor period with organ preservation and reperfusion injury or assessed the effects of prednisolone. Nevertheless this study demonstrates the clear need to consider the differential effect of donor therapies on different organ systems and the need for further research.

## Abbreviations

ADH: Antidiuretic hormone; LDH: Lactate dehydrogenase; T3: Triiodothyronine; T4: Thyroxine; AST: Aspartate aminotrasferase; ALT: Alanine aminotransferase; ACTH: Adrenocorticotropic hormone; ICAM-1: Intercellular Adhesion Molecule 1; VEGF: Vascular endothelial growth factor; vWF: Von Willebrand factor; IL-6: Interleukin 6; IL-10: Interleukin 10; TNF- α: Tumor necrosis factor; TGF- β: Transforming growth factor beta; MCP-1: Monocyte chemotactic protein-1; NF- κB: Nuclear factor kappa-light-chain-enhancer of activated B cells; HAES: Hydroxyethyl starch; NA: Noradrenaline; GAPDH: Glyceraldehyde 3-phosphate dehydrogenase; C3: Complement component 3; Bcl2: B-cell lymphoma 2; BAX: Bcl-2 associated X protein; HO-1: Heme oxygenase 1; PMNs: Polymorphonuclear neutrophils; IL-1 β: Interleukin 1 beta.

## Competing interests

The authors declare no conflict of interests.

## Authors’ contributions

RR. and LB. participated in research design, performance, data analysis and in the writing of the paper. MZA. participated in the writing of the paper. PJO. participated in the performance of the research. JNZ and RJP participated in the research design. HGL. participated in research design and in the writing of the paper. All authors read and approved the final manuscript.

## References

[B1] Bos EM Leuvenink HGD van Goor H Ploeg RJ **Kidney grafts from brain dead donors: Inferior quality or opportunity for improvement?** Kidney Int 2007 72 7 797 805 doi:10.1038/sj.ki.5002400 10.1038/sj.ki.5002400 17653138

[B2] Westendorp WH Leuvenink HG Ploeg RJ **Brain death induced renal injury** Curr Opin Organ Trans 2011 16 2 151 156 doi:10.1097/MOT.0b013e328344a5dc 10.1097/MOT.0b013e328344a5dc 21415817

[B3] Terasaki PI Cecka JM Gjertson DW Takemoto S **High survival rates of kidney transplants from spousal and living unrelated donors** N Engl J Med 1995 333 6 333 336 doi:10.1056/NEJM199508103330601 10.1056/NEJM199508103330601 7609748

[B4] Belzer FO Southard JH **Principles of solid-organ preservation by cold storage** Transplantation 1988 45 4 673 676 10.1097/00007890-198804000-00001 3282347

[B5] Maathuis M-HJ Leuvenink HGD Ploeg RJ **Perspectives in organ preservation** Transplantation 2007 83 10 1289 1298 doi:10.1097/01.tp.0000265586.66475.cc 10.1097/01.tp.0000265586.66475.cc 17519776

[B6] t Hart NA Van Der Plaats A Leuvenink HG Wiersema-Buist J Olinga P Van Luyn MJ Verkerke GJ Rakhorst G Ploeg RJ **Initial blood washout during organ procurement determines liver injury and function after preservation and reperfusion** Am J Transplant: Official J Am Soc Transplant Am Soc Transplant Surg 2004 4 11 1836 1844 10.1111/j.1600-6143.2004.00580.x 15476484

[B7] Wetzel RC Setzer N Stiff JL Rogers MC **Hemodynamic responses in brain dead organ donor patients** Anesth Analg 1985 64 2 125 128 3882020

[B8] Mascia L Bosma K Pasero D Galli T Cortese G Donadio P Bosco R **Ventilatory and hemodynamic management of potential organ donors: An observational survey*** Crit Care Med 2006 34 2 321 327 doi:10.1097/01.CCM.0000196828.87358.6E 10.1097/01.CCM.0000196828.87358.6E 16424709

[B9] Fugate JE Rabinstein AA Wijdicks EFM **Blood pressure patterns after brain death** Neurology 2011 77 4 399 401 doi:10.1212/WNL.0b013e3182270444 10.1212/WNL.0b013e3182270444 21753170

[B10] Belzberg H Shoemaker WC Wo CCJ Nicholls TP Dang ABC Zelman V Gruen JP Berne TV Demetriades D **Hemodynamic and oxygen transport patterns after head trauma and brain death: implications for management of the organ donor** J Trauma: Inj Infect Crit Care 2007 63 5 1032 1042 doi:10.1097/01.ta.0000235995.86162.d2 10.1097/01.ta.0000235995.86162.d2 17993948

[B11] Howlett TA Keogh AM Perry L Touzel R Rees LH **Anterior and posterior pituitary function in brain-stem-dead donors. A possible role for hormonal replacement therapy** Transplantation 1989 47 5 828 834 10.1097/00007890-198905000-00016 2718243

[B12] Masson F Latapie MJ Maurette P Thicoïpé M **Thyroid function in brain-dead donors** Transplant Int: Official J Eur Soc Organ Transplant 1990 3 4 226 233 10.1007/BF00366971 2076172

[B13] Fitzgerald RD Dechtyar I Templ E Pernerstorfer T Hackl W Lackner FX **Endocrine stress reaction to surgery in brain-dead organ donors** Transplant Int: Official J Eur Soc Organ Trans 1996 9 2 102 108 10.1111/j.1432-2277.1996.tb00863.x 8639250

[B14] Adrie C Monchi M Fulgencio J-P Cottias P Haouache H Alvarez-Gonzalvez A Guerrini P Cavaillon J. -M Adib-Conquy M **Immune status and apoptosis activation during brain death** Shock (Augusta, Ga) 2010 33 4 353 362, doi:10.1097/SHK.0b013e3181b65b99 10.1097/SHK.0b013e3181b65b99 20407403

[B15] Jassem W Koo DDH Cerundolo L Rela M Heaton ND Fuggle SV **Leukocyte infiltration and inflammatory antigen expression in cadaveric and living-donor livers before transplant1** Transplantation 2003 75 12 2001 2007 doi:10.1097/01.TP.0000061605.30685.03 10.1097/01.TP.0000061605.30685.03 12829901

[B16] Murugan R Venkataraman R Wahed AS Elder M Hergenroeder G Carter M Madden NJ Powner D Kellum JA **Increased plasma interleukin-6 in donors is associated with lower recipient hospital-free survival after cadaveric organ transplantation*** Crit Care Med 2008 36 6 1810 1816 doi:10.1097/CCM.0b013e318174d89f 10.1097/CCM.0b013e318174d89f 18496370

[B17] Nijboer WN Schuurs TA van der Hoeven JAB Leuvenink HGD van der Heide JJH van Goor H Ploeg RJ **Effects of brain death on stress and inflammatory response in the human donor kidney** Transplant Proc 2005 37 1 367 369 doi:10.1016/j.transproceed.2004.12.262 10.1016/j.transproceed.2004.12.262 15808646

[B18] Cooper DK Novitzky D Wicomb WN **The pathophysiological effects of brain death on potential donor organs, with particular reference to the heart** Ann R Coll Surg Engl 1989 71 4 261 266 2774455 PMC2498966

[B19] Dictus C Vienenkoetter B Esmaeilzadeh M Unterberg A Ahmadi R **Critical care management of potential organ donors: our current standard** Clin Transplant 2009 23 Suppl 21 2 9 doi:10.1111/j.1399-0012.2009.01102.x 19930309 10.1111/j.1399-0012.2009.01102.x

[B20] Shemie SD Ross H Pagliarello J Baker AJ Greig PD Brand T Cockfield S Keshavjee S Nickerson P Rao V Guest C Young K Doig C Group PR **Organ donor management in Canada: recommendations of the forum on Medical Management to Optimize Donor Organ Potential** Canadian Medical Association Journal 2006 Montréal, Que.: McGill University Health Centre 13 32 doi:10.1503/cmaj.045131 10.1503/cmaj.045131 PMC1402396 16534070

[B21] Novitzky D Cooper DKC Rosendale JD Kauffman HM **Hormonal therapy of the brain-dead organ donor: experimental and clinical studies** Transplantation 2006 82 11 1396 1401 doi:10.1097/01.tp.0000237195.12342.f1 10.1097/01.tp.0000237195.12342.f1 17164704

[B22] Damman J Hoeger S Boneschansker L Theruvath A Waldherr R Leuvenink HG Ploeg RJ Yard BA Seelen MA **Targeting complement activation in brain-dead donors improves renal function after transplantation** Transplant Immunol 2011 24 4 233 237 doi:10.1016/j.trim.2011.03.001 10.1016/j.trim.2011.03.001 21440065

[B23] Damman J Daha MR Van Son WJ Leuvenink HG Ploeg RJ Seelen MA **Crosstalk between complement and Toll-like receptor activation in relation to donor brain death and renal ischemia-reperfusion injury** Am J Transplant: Official J Am Soc Transplant Am Soc Transplant Surg 2011 11 4 660 669 doi:10.1111/j.1600-6143.2011.03475.x 10.1111/j.1600-6143.2011.03475.x 21446970

[B24] Roskott AMC Nieuwenhuijs VB Dijkstra G Koudstaal LG Leuvenink HGD Ploeg RJ **Small bowel preservation for intestinal transplantation: a review** Transplant Int: Official J Eur Soc Organ Transplant 2011 24 2 107 131 doi:10.1111/j.1432-2277.2010.01187.x 10.1111/j.1432-2277.2010.01187.x 21083772

[B25] Koudstaal LG Ottens PJ Uges DRA Ploeg RJ Van Goor H Leuvenink HGD **Increased intestinal permeability in deceased brain dead rats** Transplantation 2009 88 3 444 446 doi:10.1097/TP.0b013e3181af39bd 19667953 10.1097/TP.0b013e3181af39bd

[B26] Lam L Inaba K Branco BC Putty B Salim A Green DJ Talving P Demetriades D **The impact of early hormonal therapy in catastrophic brain-injured patients and its effect on organ procurement** Am Surg 2012 78 3 318 324 22524770

[B27] Nath DS Basha HI Liu MH Moazami N Ewald GA **Increased recovery of thoracic organs after hormonal resuscitation therapy** HEALUN 2012 29 5 594 596 doi:10.1016/j.healun.2009.12.001 10.1016/j.healun.2009.12.001 20207554

[B28] Kotsch K Ulrich F Reutzel-Selke A Pascher A Faber W Warnick P Hoffman S Francuski M Kunert C Kuecuek O Schumacher G Wesslau C Lun A Kohler S Weiss S Tullius S. G Neuhaus P Pratschke J **Methylprednisolone therapy in deceased donors reduces inflammation in the donor liver and improves outcome after liver transplantation: a prospective randomized controlled trial** Ann Surg 2008 248 6 1042 1050 doi:10.1097/SLA.0b013e318190e70c 10.1097/SLA.0b013e318190e70c 19092349

[B29] Venkateswaran RV Patchell VB Wilson IC Mascaro JG Thompson RD Quinn DW Stockley RA Coote JH Bonser RS **Early donor management increases the retrieval rate of lungs for transplantation** Ann Thorac Surg 2008 85 1 278 86286 doi:10.1016/j.athoracsur.2007.07.092 10.1016/j.athoracsur.2007.07.092 18154823

[B30] James SR Ranasinghe AM Venkateswaran R McCabe CJ Franklyn JA Bonser RS **The effects of acute triiodothyronine therapy on myocardial gene expression in brain stem dead cardiac donors** J Clin Endocrinol Metab 2010 95 3 1338 1343 doi:10.1210/jc.2009-1659 10.1210/jc.2009-1659 20080850

[B31] Macdonald PS Aneman A Bhonagiri D Jones D O’callaghan G Silvester W Watson A Dobb G **A systematic review and meta-analysis of clinical trials of thyroid hormone administration to brain dead potential organ donors*** Crit Care Med 2012 40 5 1635 1644 doi:10.1097/CCM.0b013e3182416ee7 10.1097/CCM.0b013e3182416ee7 22511141

[B32] Hodel A **Effects of glucocorticoids on adrenal chromaffin cells** J Neuroendocrinology 2001 13 2 216 220 10.1046/j.1365-2826.2001.00628.x 11168848

[B33] Rhen T Cidlowski JA **Antiinflammatory action of glucocorticoids–new mechanisms for old drugs** N Engl J Med 2005 353 16 1711 1723 doi:10.1056/NEJMra050541 10.1056/NEJMra050541 16236742

[B34] Lenth RV **Java Applets for Power and Sample Size [Computer Software]** http://homepage.stat.uiowa.edu/~rlenth/Power/

[B35] Nicolas-Robin A Barouk JD Amour J Coriat P Riou B Langeron O **Hydrocortisone supplementation enhances hemodynamic stability in brain-dead patients** Anesthesiology 2010 112 5 1204 1210 doi:10.1097/ALN.0b013e3181d4f34d 10.1097/ALN.0b013e3181d4f34d 20395825

[B36] Rech TH Moraes RB Crispim D Czepielewski MA Leitão CB **Management of the brain-dead organ donor** Transplantation 2013 95 7 966 974 doi:10.1097/TP.0b013e318283298e 10.1097/TP.0b013e318283298e 23545508

[B37] Dhar R Cotton C Coleman J Brockmeier D Kappel D Marklin G Wright R **Comparison of high- and low-dose corticosteroid regimens for organ donor management** J Crit Care 2013 28 1 111 17 doi:10.1016/j.jcrc.2012.04.015 22762934 10.1016/j.jcrc.2012.04.015

[B38] Amatschek S Wilflingseder J Pones M Kainz A Bodingbauer M Mühlbacher F Langer RM Gerlei Z Oberbauer R **The effect of steroid pretreatment of deceased organ donors on liver allograft function: A blinded randomized placebo-controlled trial** J Hepatol 2012 56 6 1305 1309 doi:10.1016/j.jhep.2012.01.020 10.1016/j.jhep.2012.01.020 22326464 PMC3355301

[B39] Bugge JF **Brain death and its implications for management of the potential organ donor** Acta Anaesthesiol Scand 2009 53 10 1239 1250 doi:10.1111/j.1399-6576.2009.02064.x 10.1111/j.1399-6576.2009.02064.x 19681785

[B40] Watson JD Varley JG Tomlin SJ Medbak S Perry L Bouloux PM Rees LH Besser GM Hinds CJ **Neuroendocrine and cardiovascular responses to high-dose corticosteroid therapy in canine endotoxin shock** Res Exp Med. Zeitschrift für die gesamte experimentelle Medizin einschliesslich experimenteller Chirurgie 1988 188 5 329 339 10.1007/BF01851201 3227157

[B41] Dahmane D Audard V Hiesse C Pessione F Bentaarit B Barrou B Rondeau E Cohen S Lang P Grimbert P **Retrospective follow-up of transplantation of kidneys from ‘marginal’ donors** Kidney Int 2006 69 3 546 552 doi:10.1038/sj.ki.5000102 10.1038/sj.ki.5000102 16407884

[B42] Ahsan N Dhillon S Holman M Yang HC **Adverse effect of donor vasopressor support on immediate and one-year kidney allograft function** Surgery 1996 120 4 663 666 10.1016/S0039-6060(96)80014-6 8862375

[B43] Bouma HR Ploeg RJ Schuurs TA **Signal transduction pathways involved in brain death-induced renal injury** Am J Transplant: Official J Am Soc Transplant Am Soc Transplant Surg 2009 9 5 989 997 doi:10.1111/j.1600-6143.2009.02587.x 10.1111/j.1600-6143.2009.02587.x 19422328

[B44] Atkinson C Varela JC Tomlinson S **Complement-dependent inflammation and injury in a murine model of brain dead donor hearts** Circ Res 2009 105 11 1094 1101 doi:10.1161/CIRCRESAHA.109.194977 10.1161/CIRCRESAHA.109.194977 19815824 PMC2783176

[B45] Damman J Nijboer WN Schuurs TA Leuvenink HG Morariu AM Tullius SG van Goor H Ploeg RJ Seelen MA **Local renal complement C3 induction by donor brain death is associated with reduced renal allograft function after transplantation** Nephrol Dial Transplant 2011 26 7 2345 2354 doi:10.1093/ndt/gfq717 10.1093/ndt/gfq717 21127132

[B46] Terry CM Clikeman JA Hoidal JR Callahan KS **TNF-**** *α* **** and IL-1**** *α* **** induce heme oxygenase-1 via protein kinase C, Ca2+, and phospholipase A2 in endothelial cells** Am J Physiol Heart Circ Physiol 1999 276 5 1493 1501 10.1152/ajpheart.1999.276.5.H1493 10330231

[B47] Hoeger S Bergstraesser C Selhorst J Fontana J Birck R Waldherr R Beck G Sticht C Seelen MA van Son WJ Leuvenink H Ploeg R Schnuelle P Yard BA **Modulation of brain dead induced inflammation by vagus nerve stimulation** Am J Transplant: Official J Am Soc Transplant Am Soc Transplant Surg 2010 10 3 477 489 doi:10.1111/j.1600-6143.2009.02951.x 10.1111/j.1600-6143.2009.02951.x 20055812

[B48] Stott B Korbelik M **Activation of complement C3, C5, and C9 genes in tumors treated by photodynamic therapy** Cancer Immunol Immunotherapy 2006 56 5 649 658 doi:10.1007/s00262-006-0221-z 10.1007/s00262-006-0221-z PMC11030087 16947020

